# Supplementary material for: Differential Expression, Tissue-Specific Distribution, and Posttranslational Controls of Phosphoenolpyruvate Carboxylase
Source: Plants (Basel). 2021 Sep 13;10(9):1887. doi: 10.3390/plants10091887 (PMC8468890; doi:10.3390/plants10091887)
Supplement: Supplementary file 1 [file plants-10-01887-s001.zip › plants-1341917-SI-.pdf]

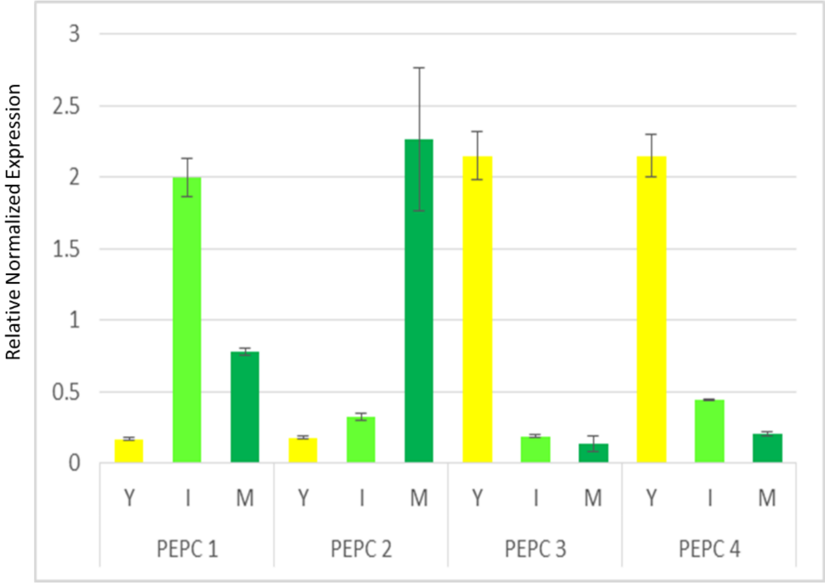

Figure S1. Relative normalized RNA expression of *B. sinuspersici* PEPC isoforms in young (Y), intermediate (I), and mature (M) leaf [17].

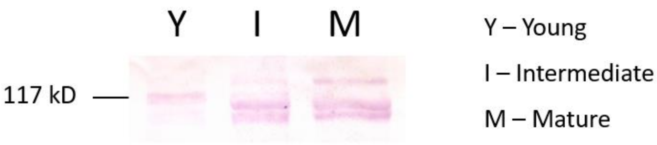

Figure S2. Immunoblot samples from young (Y), intermediate (I), and mature (M) leaf extracts showing the expression of a 117 kD BTPC [unpublished].
